# Treatment of Diphtheria

**Published:** 1861-02

**Authors:** A. Jacobi


### TREATMENT OF DIPHTHERIA.

BY A. JACOBI, M. D.

The majority of zymotic diseases require little or no medical treatment at all, especially those running their course in a distinctly typical manner. As to diphtheria, we have even made direct experiments, showing that mild cases will get well without treatment. But we think it more dangerous in diphtheria, than in other zymotic diseases, to abstain from treatment altogether, for three reasons :—Diphtheria is not a typical malady, and has a great tendency to return ; it is more of an adynamic character than any other ; and, finally, by the thick and extensive exudations of the pharynx and on the adjoining parts, it is apt to produce serious troubles, by mechanical encumbrances to deglutition and respiration. The local treatment consists of cauterization of the membranes and surrounding parts with the solid nitrate of silver, or with strong or mild solutions of the same salt in water ( ʒ ss-j. : ℥ j.) ; of gargles, consisting of solutions of (or applying in substance) astringents, such as tannic acid, alum, sulphate of zinc, or claret wine ; in gargling with, or applying, such medical agents as are known to have some effect on the constitution and tissue of the peudo-membranes, as chloride of potassium, chlorates of potassa and soda, diluted or concentrated nitric or muriatic acids, liquor of sesquichloride of iron, etc. Astringents will prevent maceration, render the exudation dry and hard, and alter the consistency of the surrounding hyperæmic and œdematous tissue. It will thus prevent, sometimes, the extension of pseudo-membranes to the neighborhood of the parts already affected, and in some cases may accelerate the expulsion of the membrane as a whole. We have thus seen the best effects from tannic acid, either applied directly to the parts by means of a curved whalebone probang, or dissolved in water as a gargle ( ʒ ss-ii. : ℥ i). Of the tinct. sesquichlor. iron we have seen no particular effect. Cauterizations with nitrate of silver we have found to be generally of very little use when applied to the pharynx. Its effect is superficial only ; it will form a scurf, but will destroy nothing. Destruction of the parts cannot be effected except by forcing the caustic into and below the membrane ; this can seldom be done in the pharynx of children, and for this reason cauterization is unavailing at this point, but will prove beneficial, we believe, by confining the process of exudation to its original locality. In cutaneous diphtheria cauterization may be exercised to its full extent, but as these cases are generally attended with extreme prostration, the general treatment will prove both more necessary and successful. If cauterization is to be resorted to, we generally use, and with good effect, more or less concentrated muriatic, or acetic, or nitro-muriatic acid. Where, however, cauterizations are made, great caution is necessary not to mistake afterwards the result of the caustic for pseudo-membrane. The remark is particularly applicable where nitrate of silver has been used.—*American Medical Times.*

